# Morphological Study of the Characteristics of Lips in Children With Mouth Breathing

**DOI:** 10.1155/ijod/1585193

**Published:** 2026-06-26

**Authors:** Lu Wenting, Wu Xiaowei, Yu Shengdan, Yan Xin, Guo Mengyuan, Hou Lili, Pan Xiaogang

**Affiliations:** ^1^ Department of Orthodontics, Shanghai Ninth Peoples’ Hospital, College of Stomatology, Shanghai Jiao Tong University School of Medicine, Shanghai Key Laboratory of Stomatology and Shanghai Research Institute of Stomatology, National Clinical Research Center for Oral Diseases, Shanghai, 200011, China, shsmu.edu.cn; ^2^ Department of Nursing, Shanghai Ninth People’s Hospital, Shanghai Jiao Tong University School of Medicine, Shanghai, 200011, China, shsmu.edu.cn

**Keywords:** children, early intervention, lip morphological characteristics, mouth breathing

## Abstract

**Objective:**

This study aimed to explore the association between long‐term mouth breathing habits and the morphological characteristics of children’s lips, providing a basis for clinical diagnosis and early intervention. Given the cross‐sectional design and reliance on parental questionnaire‐based exposure classification, causal inference is not warranted; findings are strictly associative.

**Methods:**

A total of 210 children aged 6–12 years were enrolled, comprising 105 children clinically classified as long‐term mouth‐breathing children and 105 nasal‐breathing children. Classification was based on a validated parental questionnaire with defined major and minor signs; objective airway assessment was not performed, which is acknowledged as a limitation. Lip morphological characteristics were analyzed from standardized lateral cephalograms. Quantitative parameters included protrusion of the subnasal region, upper lip, lower lip, and chin; length of the upper lip, lower lip, and chin; the ratio of upper lip length to lower lip length; nasolabial angle; mentolabial angle. Comparisons between groups used independent‐samples *t*‐tests with covariate adjustment (analysis of covariance [ANCOVA]) for age, sex, and BMI, and the Benjamini–Hochberg false discovery rate (FDR) correction for multiple comparisons. Effect sizes (Cohen’s *d*) and 95% confidence intervals (CIs) are reported.

**Results:**

Significant positive correlations were found among Ns to N′ Vert, UL to N′ Vert, LL to N′ Vert, Pog’ to N′ Vert, UL‐L, LL‐L, and Cn‐L. Compared with nasal‐breathing children, mouth‐breathing children showed decreased protrusion of the subnasal region and upper lip, retraction of the chin, a sharper nasolabial angle, and a more obtuse mentolabial angle. No significant difference in lower lip protrusion (LL to N′ Vert) was found between groups (adjusted *p* = 0.225). Because all participants were recruited from an orthodontic clinic, these findings may not be generalizable to the general pediatric population.

**Conclusions:**

Mouth breathing is associated with significant differences in lip and facial soft‐tissue morphology in children, particularly in the protrusion and length of the subnasal region, upper lip, and chin, as well as in nasolabial and mentolabial angles. Early identification and interdisciplinary management of mouth breathing may be important for preventing further morphological changes; however, longitudinal studies are needed to establish temporal relationships and causal mechanisms.

## 1. Background

Nasal breathing is the predominant pattern in daily life [1]. However, in certain situations, such as high‐intensity exercise or emotional arousal, humans naturally transition to oronasal or mouth breathing [2]. Breathing is not only a fundamental physiological requirement but also a core function of the oral and maxillofacial systems. In recent years, the high prevalence of mouth breathing in children has attracted increasing attention from orthodontists, pediatricians, and otolaryngologists [3–6].

Mouth breathing has been associated with obstructive sleep apnea, which may adversely affect growth and development, cognition, and academic performance [6–8]. Airflow through the mouth may also contribute to oral dryness, dental caries, and periodontal problems [3–5]. Long‐term respiratory dysfunction, particularly mouth breathing, has been proposed to exert a profound influence on the nasal cavity, upper respiratory tract, and overall facial structure through continuous muscular stimulation of the oral cavity and jaws during respiration [9]. A comprehensive understanding of the facial characteristics of children with mouth breathing may assist pediatricians and orthodontists in timely identification and interdisciplinary management.

Numerous studies have reported that children with lower lip‐closing force and incompetent lip seal show a higher prevalence of mouth breathing [10, 11], although it remains unclear whether incompetent lip closure is a cause or consequence of mouth breathing given the mutual influence between these conditions. Altered tongue posture and reduced tongue muscle force may also play a role; mouth‐breathing children have been found to exhibit significantly lower tongue pressure and masticatory efficiency compared with those of nasal‐breathing children [12]. These findings are consistent with Moss’s functional matrix theory, which holds that the pressure imbalance between the muscles surrounding the teeth and jaws can lead to tooth displacement and jaw shape changes, particularly during the pubertal growth spurt [13].

Childhood is a critical period for facial development, and inappropriate breathing patterns during this stage may significantly influence the facial morphology. Research indicates that fewer than 20% of children aged 3 years exhibit mouth breathing, and this proportion increases with age, reaching approximately 40% by age 12 [14]. The peak of the pubertal growth spurt occurs at around 14 years in males and 11.5 years in females [15]. Notably, the natural atrophy of adenoids partially overlaps with this critical developmental window, meaning that even after adenoid regression, its impact on facial morphology may be long‐lasting [16].

Despite the growing interest in this area, few studies have quantitatively described the specific morphological characteristics of lips in children with mouth breathing. Therefore, this study aimed to comprehensively quantify lip‐related cephalometric parameters and examine their associations with the mouth‐breathing status. It must be noted from the outset that, given the cross‐sectional design and the use of questionnaire‐based exposure classification, the findings reflect observed associations only and do not permit a causal inference.

## 2. Materials and Methods

### 2.1. Participants

The study included 210 subjects aged 6–12 years who presented for an orthodontic examination at the Department of Orthodontics, Shanghai Ninth People’s Hospital, Affiliated to Shanghai Jiao Tong University School of Medicine. The mouth‐breathing group comprised 105 children clinically classified as long‐term mouth‐breathing children (50 girls and 55 boys; mean age 9.0 ± 1.5 years). The nasal‐breathing group comprised 105 children who explicitly denied any history of mouth breathing (51 girls and 54 boys; mean age 8.9 ± 1.7 years).

Because all participants were recruited from an orthodontic clinic, the sample may not be representative of the general pediatric population, and selection bias toward children with preexisting craniofacial or occlusal concerns cannot be excluded. Replication in community‐based samples is therefore warranted.

#### 2.1.1. Definition of Long‐Term Mouth Breathing

Based on previous literature, mouth breathing was considered “long‐term” if the child had been breathing predominantly through the mouth for at least 6 months, with symptoms occurring almost every day or every night (frequency ≥5 days per week). Chronicity was determined by the parental report of persistent open‐mouth posture during wakefulness and sleep, combined with the presence of the major and minor signs described below. It is explicitly acknowledged that no objective airway assessment (e.g., rhinomanometry, nasal endoscopy, or polysomnography) was performed; reliance on questionnaire‐based classification introduces a potential risk of misclassification, particularly in children with mixed or intermittent breathing patterns.

#### 2.1.2. Orthodontic Characteristics of the Sample

Because the sample was recruited from an orthodontic clinic, the main orthodontic features were documented to characterize the sample and assess the potential selection bias. The most common reasons for seeking orthodontic evaluation were irregular teeth (45%), protruding upper incisors (28%), and concerns about jaw alignment (27%). Skeletal patterns (ANB angle) were distributed as follows: Class I (52%), Class II (34%), and Class III (14%) across the whole sample, with no significant difference between mouth‐breathing and nasal‐breathing children (*p* > 0.05).

#### 2.1.3. Inclusion Criteria (Mouth‐Breathing Children)

1. Children of both sexes aged 6–12 years.
2. According to Kogue et al. [17], two screening questions were administered: (i) “Do you often breathe through your mouth during daily activities?” and (ii) “Do you sometimes sleep with your mouth open?” A questionnaire survey was conducted on individuals who gave affirmative responses to both questions.
3. According to Valentim et al. [18], a questionnaire including major and minor signs was used to distinguish mouth‐breathing children from nasal‐breathing children. Major signs included snoring, sleeping with the mouth open, drooling on the pillow, and daily nasal congestion. Minor signs included itchy nose, sporadic nasal congestion, difficulty breathing at night or restless sleep, daytime sleepiness, daytime irritability, difficulty or delay in swallowing food, more than three episodes of throat, ear infection, or sinusitis in the previous 12 months, and difficulty in school learning or grade retention. Individuals with at least two major signs, or one major sign and two minor signs, were classified as mouth‐breathing children. Individuals with no more than one major sign occurring occasionally and/or one minor sign were classified as nasal‐breathing children.
4. All children had no obvious systemic diseases or congenital deformities of the lips, mouth, or nose.
5. All participants’ legal guardians signed an informed consent form drafted in accordance with the Declaration of Helsinki.

#### 2.1.4. Exclusion Criteria

1. A history of chronic respiratory diseases (e.g., asthma and chronic sinusitis).
2. A history of lip or oral surgery.
3. Recent use of medications that may affect lip or oral muscle condition (e.g., long‐term corticosteroid use).
4. Severe psychological or behavioral disorders.

### 2.2. Data Collection

Lateral cephalograms were obtained with the participant in a natural head position (mirror‐guided), lips relaxed in a habitual resting posture, and teeth in centric occlusion. All radiographs were taken while the participant breathed quietly through the nose (or mouth, if unable) to simulate natural conditions. The same X‐ray device (Siemens, Germany) was used with a source‐to‐film distance of 150 cm and a film‐to‐mid‐sagittal‐plane distance of 15 cm, resulting in a uniform magnification factor of 11.1%, which was corrected within the Dolphin Imaging software (version 11.95, Dolphin Imaging and Management Solutions, Chatsworth, CA, USA).

**Figure 1:**
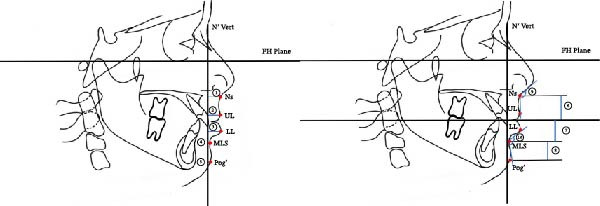
Cephalometric landmarks and reference lines. Reference lines include the Frankfort horizontal (FH) plane and the González–Ulloa meridian line (N′ Vert), which is perpendicular to the FH plane. All linear measurements represent horizontal distances perpendicular to the N′ Vert line. Landmarks: Ns, subnasal point; UL, upper lip; LL, lower lip; MLS, deepest point of the mentolabial sulcus; Pog’, soft‐tissue pogonion. Measurements: ①Ns to N′ Vert, ②UL to N′ Vert, ③LL to N′ Vert, ④MLS to N′ Vert, ⑤Pog’ to N′ Vert, ⑥UL‐L, ⑦LL‐L, ⑧Cn‐L, ⑨NL‐A, and ⑩ML‐A.

Digital tracings were performed using the Dolphin Imaging Software. The horizontal reference line was defined as the Frankfort horizontal (FH) plane, and the vertical reference line was defined as the González–Ulloa meridian line (N′ Vert), a perpendicular line to the FH plane passing through soft‐tissue nasion. Cephalometric measurements included protrusion of the subnasal region, upper lip, lower lip, and chin; thickness at the mentolabial sulcus; length of the upper lip, lower lip, and chin; the ratio of upper lip length to lower lip length; nasolabial angle (NL‐A); mentolabial angle (ML‐A; Table 1 and Figure 1). All linear measurements were made as horizontal distances to the N′ Vert line (perpendicular to the FH plane) unless otherwise specified. Angular measurements were defined, as described in Table 1.

**Table 1:** Definition of the cephalometric measurements.

Measurement	Definition
The protrusion of the subnasal (Ns to N′ Vert)	Vertical distance from the subnasal point to the N′ vert line
The protrusion of the upper lip (UL to N′ Vert)	Vertical distance from the most prominent point of the upper lip to the N′ vert line
The protrusion of the lower lip (LL to N′ Vert)	Vertical distance from the most prominent point of the lower lip to the N′ vert line
The thickness at mentolabial sulcus (MLS to N′ Vert)	Vertical distance from the deepest point of the mentolabial sulcus to the N′ vert line
The protrusion of the chin (Pog’ to N′ Vert)	Vertical distance from the pogonion of soft tissue to the N′ vert line
The length of the upper lip (UL‐L)	Translate the FH plane to the contacting point between the upper and lower lips, and draw a perpendicular line from the subnasal point to that line
The length of the lower lip (LL‐L)	Translate the FH plane to the contacting point between the upper and lower lips, and draw a perpendicular line from the deepest point of the mentolabial sulcus to that line
The ratio of UL‐L to LL‐L (UL‐L/LL‐L)	The ratio of the upper lip length to the lower lip length
The length of the chin (Cn‐L)	Translate the FH plane across pogonion, and draw a perpendicular line from the deepest point of the mentolabial sulcus to that line
The nasolabial angle (NL‐A)	The anterior intersection angle of the line connecting the subnasal point and the nasal columella point, and the line connecting the subnasal point and the most prominent point of the upper lip
The mentolabial angle (ML‐A)	The anterior intersection angle between the line connecting the deepest point of the mentolabial sulcus and the most prominent point of the lower lip, and the tangent line passing through the deepest point of mentolabial sulcus to the chin

### 2.3. Statistical Analysis

Statistical analysis was conducted using SPSS version 24.0 (IBM Corp., Armonk, NY, USA). Before parametric tests, normality was assessed using the Shapiro–Wilk test and homogeneity of variances using Levene’s test for all continuous variables; all variables met these assumptions (*p* > 0.05). Baseline comparisons between groups were performed using independent‐samples *t*‐tests for continuous variables and chi‐square tests for categorical variables; exact test statistics and *p* values are reported in Table 2. Differences were defined as statistically significant if  ^∗^ *p* < 0.05,  ^∗∗^ *p* < 0.01,  ^∗∗∗^ *p* < 0.001.

**Table 2:** Summary statistics of the cephalometric measurements.

Measurements	Mouth breathing group(*n* = 105)	Nasal breathing group(*n* = 105)	*p* value
Age (years)	9 ± 1.5	8.9 ± 1.7	>0.05
Sex (male/female)	55/50	54/51	>0.05
BMI	17.2 ± 2.1	16.8 ± 1.9	0.02 ^∗^

Correlation analysis was performed using Pearson correlation coefficients (Table 3). To compare lip morphological characteristics between mouth‐breathing and nasal‐breathing children, independent‐sample *t*‐tests were first conducted. Because BMI differed significantly between groups (*t* = 2.34, *p* = 0.020) and is a potential confounder, analysis of covariance (ANCOVA) was additionally performed adjusting for age, sex, and BMI. Adjusted estimates, 95% confidence intervals (CIs), and Cohen’s *d* effect sizes are reported in Table 4. To account for multiple comparisons, the Benjamini–Hochberg false discovery rate (FDR) correction was applied, with a significance threshold of *q* < 0.05. Both unadjusted and FDR‐corrected *p* values are presented in Table 4.

**Table 3:** Pearson correlation analysis between different lip morphological features.

Significance (*p*)		Correlation coefficient (*r*)						
		UL to N′ Vert (mm)	MLS to N′ Vert (mm)	LL to N′ Vert (mm)	Pog’ to N′ Vert (mm)	UL‐L (mm)	LL‐L (mm)	Cn‐L (mm)
Ns to N′ Vert(mm)	*r* = 0.82, *p* < 0.001		*r* = 0.71, *p* < 0.001	*r* = 0.76, *p* < 0.001	*r* = 0.63, *p* < 0.01	*r* = 0.67, *p* < 0.001	*r* = 0.79, *p* < 0.001	*r* = 0.74, *p* < 0.001
UL to N′ Vert (mm)	—		*r* = 0.59, *p* < 0.001	*r* = 0.84, *p* < 0.001	*r* = 0.65, *p* < 0.01	*r* = 0.72, *p* < 0.001	*r* = 0.68, *p* < 0.001	*r* = 0.70, *p* < 0.001
MLS to N′ Vert (mm)	*r* = 0.59, *p* < 0.001		—	*r* = 0.63, *p* < 0.001	*r* = 0.75, *p* < 0.001	*r* = 0.58, *p* < 0.001	*r* = 0.61, *p* < 0.001	*r* = 0.69, *p* < 0.001
LL to N′ Vert (mm)	*r* = 0.84, *p* < 0.001		*r* = 0.63, *p* < 0.001	—	*r* = 0.68, *p* < 0.001	*r* = 0.64, *p* < 0.001	*r* = 0.77, *p* < 0.001	*r* = 0.72, *p* < 0.001
UL‐L (mm)	*r* = 0.65, *p* < 0.01		*r* = 0.75, *p* < 0.001	*r* = 0.68, *p* < 0.001	*r* = 0.62, *p* < 0.001	—	*r* = 0.65, *p* < 0.001	*r* = 0.71, *p* < 0.001
LL‐L (mm)	*r* = 0.72, *p* < 0.001		*r* = 0.58, *p* < 0.001	*r* = 0.64, *p* < 0.001	*r* = 0.62, *p* < 0.001	*r* = 0.71, *p* < 0.001	—	*r* = 0.69, *p* < 0.001
Cn‐L (mm)	*r* = 0.70, *p* < 0.001		*r* = 0.69, *p* < 0.001	*r* = 0.72, *p* < 0.001	*r* = 0.71, *p* < 0.001	*r* = 0.68, *p* < 0.001	*r* = 0.73, *p* < 0.001	—
UL‐L/LL‐L (%)	*r* = 0.45, *p* < 0.01		*r* = 0.37, *p* < 0.05	*r* = 0.51, *p* < 0.001	*r* = 0.39, *p* < 0.01	*r* = 0.43, *p* < 0.001	*r* = 0.54, *p* < 0.001	*r* = 0.46, *p* < 0.001
NL‐A (°)	*r* = −0.41, *p* < 0.01		*r* = −0.32, *p* < 0.05	*r* = −0.46, *p* < 0.001	*r* = −0.35, *p* < 0.01	*r* = −0.38, *p* < 0.01	*r* = −0.49, *p* < 0.001	*r* = −0.42, *p* < 0.001

**Table 4:** Comparison of lip morphological characteristics between mouth and nasal breathing groups.

Measurements	Mouth breathing children (*n* = 105)	Nasal breathing children (*n* = 105)	Mean difference (95% CI)	Cohen’s *d*	Significance (*q*)
Ns to N′ Vert (mm)	8.2 ± 1.5	10.3 ± 1.2	−2.1 (–2.7 to –1.5)	1.53	<0.001
UL to N′ Vert (mm)	4.5 ± 0.8	5.8 ± 0.9	−1.3 (–1.7 to –0.9)	1.53	<0.001
MLS to N′ Vert (mm)	2.9 ± 0.7	2.1 ± 0.5	0.8 (0.5–1.1)	1.29	<0.001
LL to N′ Vert (mm)	5.3 ± 0.9	5.5 ± 0.8	−0.2 (–0.5 to 0.1)	0.23	NS
Pog’ to N′ Vert (mm)	11.4 ± 1.6	13.2 ± 1.5	−1.8 (–2.4 to –1.2)	1.17	<0.001
UL‐L (mm)	17.6 ± 1.8	16.2 ± 1.7	1.4 (0.7–2.1)	0.80	0.003
LL‐L (mm)	18.3 ± 1.9	17.5 ± 1.8	0.8 (0.2–1.4)	0.44	0.067 (marginal)
Cn‐L (mm)	19.1 ± 2.0	21.0 ± 1.9	−1.9 (–2.6 to –1.2)	0.99	<0.001
UL‐L/LL‐L (%)	0.96 ± 0.04	0.93 ± 0.03	0.03 (0.01–0.05)	0.86	0.005
NL‐A (°)	98.5 ± 4.2	102.3 ± 3.8	−3.8 (–5.3 to –2.3)	0.96	<0.001
ML‐A (°)	120.2 ± 5.4	117.1 ± 4.9	3.1 (1.2–5.0)	0.60	0.005

Two examiners (Lu Wenting and Wu Xiaowei) independently traced all cephalograms after a calibration session. Each examiner traced 20 randomly selected radiographs twice, with a 2 week interval between sessions, to assess intra‐examiner and inter‐examiner reliability. Intraclass correlation coefficients (ICC) with 95% CIs were calculated using a two‐way mixed‐effects model (absolute agreement). Measurement error was estimated using the Dahlberg’s formula. ICC values for all cephalometric variables exceeded 0.90, indicating excellent reliability.

## 3. Results

A total of 210 participants satisfied the selection criteria: 105 mouth‐breathing children (50 females and 55 males) and 105 nasal‐breathing children (51 females and 54 males). The mean age was 9.0 ± 1.5 years in the mouth‐breathing group and 8.9 ± 1.7 years in the nasal‐breathing group (*t* = 0.46, *p* = 0.645). The mean BMI was slightly but significantly higher in mouth‐breathing children (17.2 ± 2.1) than in nasal‐breathing children (16.8 ± 1.9; *t* = 2.34, *p* = 0.020; Table 2). Given the modest absolute mean difference in BMI (0.4 kg/m^2^), this difference, while statistically significant, may be of limited clinical magnitude; BMI was nonetheless retained as a covariate in all adjusted analyses.

### 3.1. Reliability Results

Intra‐examiner ICCs ranged from 0.92 to 0.98 (95% CI: 0.88–0.99) across all measurements. Inter‐examiner ICCs ranged from 0.90 to 0.97 (95% CI: 0.85–0.98). The Dahlberg errors were below 0.5 mm for linear measurements and below 1.5° for angular measurements, indicating high reproducibility.

### 3.2. Correlation Analysis

Pearson correlation analysis revealed significant correlations among different lip and facial morphological parameters (Table 3). Key findings are as follows:

1. Ns to N′ Vert was positively correlated with UL to N′ Vert and other facial features, especially LL to N′ Vert (*r* = 0.76, *p* < 0.001) and LL‐L (*r* = 0.79, *p* < 0.001).
2. UL to N′ Vert was strongly positively correlated with LL to N′ Vert (*r* = 0.84, *p* < 0.001), indicating consistency in upper and lower lip prominence.
3. MLS to N′ Vert was most strongly correlated with Pog’ to N′ Vert (*r* = 0.75, *p* < 0.001).
4. LL to N′ Vert was closely related to Ns to N′ Vert and also positively correlated with Pog’ to N′ Vert (*r* = 0.68, *p* < 0.001), reflecting uniformity of the lower facial structure.
5. Pog’ to N′ Vert showed significant positive correlations with most facial parameters.
6. A moderate‐to‐strong correlation (*r* = 0.69, *p* < 0.001) between UL‐L and LL‐L indicated coordination of lip lengths.
7. The correlation between LL‐L and Cn‐L (*r* = 0.73, *p* < 0.001) was particularly prominent, reflecting coherence of the mandibular region.
8. UL‐L/LL‐L was moderately positively correlated with multiple parameters including lip protrusion and length, especially LL to N′ Vert (*r* = 0.51, *p* < 0.001) and LL‐L (*r* = 0.54, *p* < 0.001).
9. NL‐A was mildly to moderately negatively correlated with these parameters (*r* from −0.41 to −0.49), particularly influenced by LL to N′ Vert and LL‐L.

### 3.3. Between‐Group Comparisons (*t*‐Tests and ANCOVA)

Table 4 presents the results of independent *t*‐tests and ANCOVA adjusting for age, sex, and BMI, along with FDR‐corrected *p* values. Both unadjusted and adjusted significance levels are distinguished in the table. After FDR correction, all significant differences were maintained except for LL‐L (adjusted *p* = 0.044), which was considered marginally significant and did not survive FDR correction (*q* = 0.067).

1. Ns to N′ Vert (mm): Mouth‐breathing children: 8.2 ± 1.5; nasal‐breathing children: 10.3 ± 1.2. MD = −2.1 (95% CI: −2.7 to −1.5), Cohen’s *d* = 1.53, adjusted *p* < 0.001, *q* < 0.001.
2. UL to N′ Vert (mm): Mouth‐breathing children: 4.5 ± 0.8; nasal‐breathing children: 5.8 ± 0.9. MD = −1.3 (95% CI: −1.7 to −0.9), *d* = 1.53, adjusted *p* < 0.001, *q* < 0.001.
3. MLS to N′ Vert (mm): Mouth‐breathing children: 2.9 ± 0.7; nasal‐breathing children: 2.1 ± 0.5. MD = 0.8 (95% CI: 0.5–1.1), *d* = 1.29, adjusted *p* < 0.001, *q* < 0.001.
4. LL to N′ Vert (mm): Mouth‐breathing children: 5.3 ± 0.9; nasal‐breathing children: 5.5 ± 0.8. MD = −0.2 (95% CI: −0.5 to 0.1), *d* = 0.23, adjusted *p* = 0.225, *q* = 0.225. Not significant.
5. Pog’ to N′ Vert (mm): Mouth‐breathing children: 11.4 ± 1.6; nasal‐breathing children: 13.2 ± 1.5. MD = −1.8 (95% CI: −2.4 to −1.2), *d* = 1.17, adjusted *p* < 0.001, *q* < 0.001.
6. UL‐L (mm): Mouth‐breathing children: 17.6 ± 1.8; nasal‐breathing children: 16.2 ± 1.7. MD = 1.4 (95% CI: 0.7–2.1), *d* = 0.80, adjusted *p* = 0.001, *q* = 0.003.
7. LL‐L (mm): Mouth‐breathing children: 18.3 ± 1.9; nasal‐breathing children: 17.5 ± 1.8. MD = 0.8 (95% CI: 0.2–1.4), *d* = 0.44, adjusted *p* = 0.044, *q* = 0.067. Marginally significant; did not survive FDR correction.
8. Cn‐L (mm): Mouth‐breathing children: 19.1 ± 2.0; nasal‐breathing children: 21.0 ± 1.9. MD = −1.9 (95% CI: −2.6 to −1.2), *d* = 0.99, adjusted *p* < 0.001, *q* < 0.001.
9. UL‐L/LL‐L (%): Mouth‐breathing children: 0.96 ± 0.04; nasal‐breathing children: 0.93 ± 0.03. MD = 0.03 (95% CI: 0.01–0.05), *d* = 0.86, adjusted *p* = 0.002, *q* = 0.005.
10. NL‐A (°): Mouth‐breathing children: 98.5 ± 4.2; nasal‐breathing children: 102.3 ± 3.8. MD = −3.8 (95% CI: −5.3 to −2.3), *d* = 0.96, adjusted *p* < 0.001, *q* < 0.001.
11. ML‐A (°): Mouth‐breathing children: 120.2 ± 5.4; nasal‐breathing children: 117.1 ± 4.9. MD = 3.1 (95% CI: 1.2–5.0), *d* = 0.60, adjusted *p* = 0.002, *q* = 0.005.

## 4. Discussion

The current study revealed complex interrelationships among lip and facial soft tissue morphological parameters through Pearson correlation analysis. The significant correlations indicate that lip morphology parameters do not exist in isolation but interact and constrain one another. For example, the positive correlation between Ns to N′ Vert and UL to N′ Vert, LL to N′ Vert, Pog’ to N′ Vert, UL‐L, LL‐L, and Cn‐L suggests that greater subnasal prominence is often accompanied by synchronous protrusion of the lips and chin, which may relate to the coordinated development of the alveolar bone and soft tissue.

Comparison between mouth‐breathing and nasal‐breathing children showed significant differences in multiple lip characteristics after adjustment for age, sex, and BMI. A notable finding was the absence of a significant difference in lower lip protrusion (LL to N′ Vert; adjusted *p* = 0.225), indicating that the horizontal position of the lower lip relative to the N′ Vert line is not meaningfully associated with mouth‐breathing status in this sample. This is contrary to some prior suggestions of generalized lower lip retrusion (e.g., [19]). The main differences observed were in the upper lip, chin, and angular measurements: mouth‐breathing children exhibited a more retrusive upper lip and chin, a sharper nasolabial angle, and a more obtuse mentolabial angle. These patterns are consistent with the known tendency for children with mouth breathing to exhibit retrognathic features and a clockwise mandibular rotation [20–24].

The absence of a significant difference in LL to N′ Vert may be explained by compensatory mechanisms: although the mandible may be retrognathic, the lower lip may sometimes evert to maintain contact with the upper lip, particularly in children with an increased overjet. Alternatively, lower lip thickness may be preserved through muscular adaptation [25]. Importantly, the absence of a significant difference does not imply that the lower lip is entirely unaffected by mouth breathing; rather, its horizontal protrusion relative to the N′ Vert line may be less sensitive to the mouth‐breathing status than other measured parameters. Three‐dimensional imaging studies could provide complementary insights.

### 4.1. Residual Confounding and Methodological Considerations

Although the adjusted analyses accounted for age, sex, and BMI, residual confounding from unmeasured factors cannot be excluded. Important potential confounders include the growth stage (pubertal maturation), skeletal pattern, malocclusion severity, lip competence, and the underlying etiology of mouth breathing. These variables were not systematically controlled and may have independently influenced the observed morphological differences. Future studies should incorporate measures of these factors, ideally using a community‐based longitudinal design.

The use of questionnaire‐based exposure classification without objective airway assessment (rhinomanometry, nasal endoscopy, or polysomnography) represents the most significant methodological limitation of this study. Children with mixed or intermittent breathing patterns may have been misclassified, attenuating or distorting the observed associations. Additionally, because all participants were recruited from an orthodontic clinic, the sample likely over‐represents children with preexisting craniofacial concerns, limiting the external validity of these findings. These limitations substantially restrict the strength of the conclusions that can be drawn.

The etiology of mouth breathing is heterogeneous, spanning obstructive causes (e.g., adenotonsillar hypertrophy and allergic rhinitis) and functional or habitual causes. This study did not differentiate between etiological subgroups. Future research should be stratified by etiology to determine whether specific causes are associated with distinct morphological patterns.

### 4.2. Implications

Despite these limitations, the findings suggest that mouth‐breathing children may exhibit characteristic soft‐tissue facial differences relative to those of nasal‐breathing children, including retrusion of the upper lip and chin, a sharper nasolabial angle, and a more obtuse mentolabial angle. Clinicians should be aware that reliance on lower lip position alone may be misleading as lower lip protrusion was not significantly associated with mouth‐breathing status in this study. Interdisciplinary collaboration among orthodontists, pediatricians, and otolaryngologists is important for comprehensive evaluation and management. Because this is a cross‐sectional, associative study, we do not advocate specific clinical interventions based on these results alone. Longitudinal studies tracking children from before the onset of mouth breathing through development are needed to clarify the causal mechanisms and to identify critical windows for intervention.

## 5. Conclusion

This study found that mouth breathing is associated with significant differences in lip soft‐tissue morphology in children, particularly in the protrusion and length of the subnasal region, upper lip, and chin, as well as in nasolabial and mentolabial angles. Specifically, compared with nasal‐breathing children, mouth‐breathing children showed a more retrusive upper lip and chin, a sharper nasolabial angle, and an increased mentolabial angle. Lower lip protrusion was not significantly different between the groups. Because this study is cross‐sectional and exposure classification relied on parental questionnaires without objective airway measurement, these findings reflect associations and should not be interpreted causally. The sample was also drawn from an orthodontic clinic, limiting generalizability to the broader pediatric population. These findings enrich our understanding of the relationship between mouth breathing and lip morphology and provide a basis for clinical awareness and further research. Future studies should employ longitudinal designs, objective exposure classification, and community‐based samples to establish temporal relationships and improve generalizability.

## Author Contributions

Lu Wenting and Wu Xiaowei contributed to the investigation, analysis, original draft preparation, and manuscript review and editing. Yu Shengdan, Yan Xin, and Guo Mengyuan contributed to the review and editing of the manuscript. Pan Xiaogang and Hou Lili contributed to the conceptualization, project administration, validation, and manuscript reviewing and editing.

## Funding

This study was funded by the Nursing Discipline Development Fund of the Shanghai Ninth Peoples’ Hospital (Grant JYHL2024ZD02‐Z2).

## Ethics Statement

The protocol was approved by the Institutional Research Ethics Committee of Shanghai Ninth Peoples’ Hospital (SH9H‐2019‐T305‐9). Informed consent was obtained from all subjects or their legal guardians. We ensure that the study was carried out in accordance with the Code of Ethics of the World Medical Association (Declaration of Helsinki).

## Consent

The authors have nothing to report.

## Conflicts of Interest

The authors declare no conflicts of interest.

## Data Availability

The data used and/or analyzed during the current study are available from the corresponding author upon reasonable request.
