# Correction to “Morphological Study of the Characteristics of Lips in Children With Mouth Breathing”

**DOI:** 10.1155/ijod/9769151

**Published:** 2026-08-11

L. Wenting, W. Xiaowei, Y. Shengdan, et al., “Morphological Study of the Characteristics of Lips in Children With Mouth Breathing,” *International Journal of Dentistry* 2026 (2026): 1585193, https://doi.org/10.1155/ijod/1585193.

Correction to Affiliation for First Author Lu Wenting:

1. School of Nursing, Shanghai Jiao Tong University, Shanghai 200025, China

Then, the following author superscripts and affiliation numbers will be changed.

Wu Xiaowei^2^, Yu Shengdan^2^, Yan Xin^2^, Guo Mengyuan^2^, Pan Xiaogang^2^

2. Department of Orthodontics, Shanghai Ninth People’s Hospital, College of Stomatology, Shanghai Jiao Tong University School of Medicine, National Clinical Research Center for Oral Diseases, Shanghai Key Laboratory of Stomatology & Shanghai Research Institute of Stomatology, Shanghai 200011, China

Hou Lili^3^

3. Department of Nursing, Shanghai Ninth People’s Hospital, Shanghai Jiao Tong University School of Medicine, Shanghai 200011, China

We apologize for this error.

